# 927 Battle of the Sexes: Opioid Consumption Comparisons Between Male and Female Burn Victims

**DOI:** 10.1093/jbcr/iraf019.458

**Published:** 2025-04-01

**Authors:** Reaksmey San, Maila Rodriguez, Matthew Reiss, Connie Van, Felix Pham

**Affiliations:** Torrance Memorial Medical Center; Torrance Memorial Medical Center; Torrance Memorial Medical Center; Torrance Memorial Medical Center; Torrance Memorial Medical Center

## Abstract

**Introduction:**

Burn injuries pose significant challenges due to complex treatments and the need for effective pain management. Opioid consumption in burn patients is influenced by factors such as prior opioid use, Total Body Surface Area (TBSA) affected, weight and other patient-specific variables. This study explores sex characteristics as a potential factor in opioid use, aiming to improve tailored strategies for pain management.

While existing literature indicates that sex may affect both opioid use and pain perception, with females generally showing higher pain sensitivity, limited data specifically addresses its role in burn patients. This study seeks to determine whether sex characteristics impact opioid consumption in this population.

**Methods:**

A retrospective analysis was conducted using patient charts from a burn unit and data from the national burn database for the fiscal year 2023-2024. The study included patients aged 18 years and older, with burns 5% or greater TBSA, and hospital stays of over 24 hours. Patients were stratified by TBSA into four brackets (5-10%, >10-15%, >15-20%, and >20%) for comparison.

Opioid consumption data was collected and converted into morphine milligram equivalents (MME). Each patient’s total opioid use was calculated as MME per hospital length of stay, normalized by body weight (MME/kg/day). A two-tailed, unpaired Student’s t-test was used to compare opioid consumption between males and females, with a significance level of 0.05. Patients with a history of substance abuse were excluded from the study.

**Results:**

The study included 25 females and 38 males. No statistically significant differences were found in opioid consumption between genders when stratified by TBSA, with each bracket having a p-value greater than 0.05.

**Conclusions:**

Sex characteristics were not associated with differences in opioid consumption in this sample of burn patients. However, our research was underpowered and further research with larger sample sizes may improve our analysis of potential differences more conclusively. At present, sex characteristics should not be a primary factor in opioid prescribing for burn victims until more evidence is available.

**Applicability of Research to Practice:**

This study underscores the under-investigated examination of sex differences in burn treatment. While no significant differences were observed in this study, the methods may help to normalize comparison of opioid use for future studies, where larger populations would enhance the understanding of sex-based differences in opioid consumption.

**Funding for the Study:**

The authors have received no financial support for the research, authorship, and/or publication of this paper.

